# A prospective assessment of gait kinematics and related clinical examination measures in cerebral palsy crouch gait

**DOI:** 10.12688/hrbopenres.13647.1

**Published:** 2022-12-06

**Authors:** Rory O'Sullivan, Helen French, Frances Horgan

**Affiliations:** 1 Specialist Services, Central Remedial Clinic, Dublin, Ireland; 2 School of Physiotherapy, Royal College of Surgeons in Ireland, Dublin, Ireland

**Keywords:** cerebral palsy, crouch gait, knee flexion, kinematics, gait analysis

## Abstract

Background

While prospectively assessed crouch gait in cerebral palsy (CP) does not necessarily progress, prospective changes in clinical examination measures have not been reported.

This study prospectively examined the association between selected clinical examination variables and change in crouch gait in a cohort with bilateral CP.

Methods

Inclusion criteria were a diagnosis of ambulant bilateral CP, knee flexion at mid-stance >19 ^0^ and a minimum of two-years between gait analyses. The change in kinematic variables was assessed using Statistical Parameter Mapping (SPM) and changes in clinical measures using appropriate paired tests. Linear regression examined the association between progression of crouch and clinical examination variables.

Results

There was no mean change in crouch in 27 participants over 3.29 years. However, there was significant variability within this group. Clinical hamstring tightness (60.00 ^0^ to 70.48 ^0^, p<0.01) and external knee rotation during stance (SPM analysis, p<0.001) increased but there was no association between changes in clinical examination variables and changes in crouch (p-values 0.06 - 0.89).

Conclusions

The variability crouch gait progression highlights the pitfall of group mean values in such a heterogeneous population.  The lack of association between changes in clinical examination variables and changes in crouch highlights the multi-factorial aetiology of this gait pattern and the need for larger prospective studies.

## Introduction

In cerebral palsy (CP), crouch gait and flexed knee gait are largely synonymous terms referring to excessive knee flexion in stance phase ^1^ and are among the most common pathological gait patterns in CP ^2^. A systematic review on the progression of crouch gait in CP suggested that, in the absence of surgical intervention, knee flexion progresses over time ^3^. However, our recent prospective follow-up of crouch gait in CP ^4^ found that crouch gait does not necessarily progress over time and on assessment every six-months, participants demonstrated episodes of both increasing and decreasing crouch. These contrasting findings appeared to be a function of prospective versus retrospective study designs. The majority of the studies included in the systematic review were retrospective cohort studies ^5– 8^) likely based on data collected from those referred for clinical gait analysis. As referral for clinical gait analysis is often as a result of a deterioration in function, pain or to aid with planning of required intervention, it is not surprising, therefore, that retrospective cohort studies based on such data demonstrate deterioration in gait between repeated analyses. The need for clinically based prospective studies of walking in CP has previously been highlighted ^9^ and the findings of our recent prospective study of crouch gait ^4^ supports this. That prospective study focussed only on the changes in knee flexion at mid-stance every six months and did not report on contemporaneous changes in other relevant gait kinematic variables or clinical examination measures. This was largely because the clinical examination variables in particular were unlikely to change sufficiently over six-monthly assessments to allow meaningful analysis, particularly when considering measurement error. The current study aims to examine the longer-term changes in crouch gait and the associated changes in relevant clinical examination and kinematic variables.

The most relevant kinematic and clinical examination variables are those potentially implicated in the causation of couch gait which is acknowledged to be multifactorial and due to a combination of muscular, neurologic, and/or bony pathologic processes ^10^. Tightness in the knee flexors has been implicated, and crouch is often treated with surgical release of the hamstrings ^11, 12^. However, muscle-length modelling has suggested that most subjects with crouch gait have hamstrings of normal length or longer ^13^. While it has previously been suggested that hamstring tighten secondary to crouch gait rather than causing this gait pattern ^3^, this was based on retrospectively analyzed gait data and a prospective examination of the relationship between crouch gait and hamstring tightness has not previously been reported. Dynamically, during gait, external tibial torsion has been shown to reduce the capacity of muscles to extend both the hip and knee ^14^. Therefore, lever arm dysfunction (LAD), which refers to faulty skeletal alignment ^15^, is thought to contribute to crouch gait. Excessive external tibial torsion with excessive internal hip rotation due to femoral anteversion is the classic form of LAD in CP ^16^.

The aim of this study was to examine the prospective progression of crouch gait over a minimum of two years along with the contemporaneous changes in clinical examination measures of muscle tightness and bony torsions in a cohort of ambulant participants with CP crouch gait and to assess the association between progression in crouch gait and these variables.

## Methods

Approval for the study was obtained from the Research Ethics Committee of the Central Remedial Clinic and written, informed consent was obtained from all parents/guardians. Participant recruitment and kinematic data collection methodology were as previously described ^4^. Inclusion criteria were a diagnosis of bilateral, spastic CP, GMFCS level I-III, age 4–17 years at first analysis and crouch gait. Crouch gait was defined as knee flexion greater or equal to two standard deviations above laboratory reference values at mid-stance in at least one limb. This equated to mid-stance knee flexion greater or equal to 19°. Participants were excluded if they had surgery within one year prior to the first gait analysis. Sex was as recorded in the participants’ medical file.

Kinematic data were captured using a four camera Codamotion cx1 active marker system (Charnwood Dynamics, Leicestershire, UK) at a rate of 200 Hz using a modified Helen Hayes marker protocol ^17^. A dedicated, multisegment foot model was not used in this study and the foot was modelled as a straight-line segment from the base of the fifth metatarsal to the lateral aspect of the calcaneus. Therefore, only sagittal plane foot data (dorsiflexion/plantarflexion) were analysed. All participants walked in barefoot at a self-selected speed. Participants walked independently where possible. If independent gait was not possible data were collected while the participant walked with the assistance of two hands held in front by a physiotherapist as is standard practice in the gait laboratory. A minimum of four walking trials was recorded for each participant and one representative walking trial was chosen for further analysis.

Additional to the previously described barefoot kinematic data collection procedures, relevant clinical examination measures of muscle tightness/contracture and bony torsions were collected, and all data collection (kinematic and clinical) was by the same examiner on each occasion. Popliteal angle measure of clinical hamstring tightness was recorded in supine with the contralateral hip and knee flexed to align the pelvis to neutral, the recorded measure was degrees from full extension. Hip flexion contracture was similarly assessed in supine with the pelvis in neutral. The hip was passively extended to the limit of motion and the recorded value was lack of extension to neutral. Knee flexion contracture was also recorded in supine as lack of full extension. Gastrocnemius tightness was assessed with the knee extended and recorded as the angle between the longitudinal line of the tendo-achilles and the longitudinal axis of the calcaneus. Soleus tightness was similarly measured with the knee flexed. Femoral torsion was estimated in prone lying using the trochanteric prominence angle test ^18^. Tibial torsion was estimated by measuring the transmalleolar angle corresponding to the angle between the line connecting the medial and lateral malleoli and a line perpendicular to the long axis of the thigh ^19, 20^.

From the previously described cohort ^4^, those who had a minimum of two years between their first and last assessments were identified and the changes in relevant kinematic and clinical examination variables between those two assessments were examined. A number of participants progressed to surgical intervention during the course of the study and so did not have two years between their first and last pre-operative assessments. This group were not included in the present analysis but their data are included in the Extended Data for completeness (Figure S.2; Table S.1).

To avoid dependence between sides, only the side which was most flexed during gait at first assessment was chosen for subsequent analysis ^12, 21^. All clinical examination variables reported were first tested for normality using a Shapiro-Wilks test. Normally distributed data were summarised using means and standard deviations. Otherwise, medians and inter-quartile ranges were reported. Differences in clinical examination variables were assessed using paired t-tests for normally distributed data. Otherwise, Wilcoxon Signed-Ranks tests were used. The presence of a hip/knee flexion contracture was defined as a clinically measured contracture ≥ 5° and changes in the prevalence of flexion contractures were assessed using Pearson’s chi-squared test. Significance was set at p<0.05 in all cases.

Gait kinematic data were analysed using Statistical Parametric Mapping (SPM) (SPM1d version 0.4, available for download at http://www.spm1d.org/) in MATLAB (The Mathworks Inc., Natick, M.A., 2015). Significance level for all analyses was set at p<0.05.

The association between the progression of crouch gait and relevant clinical examination variables was assessed using repeated liner regression using each relevant demographic or clinical examination variable as a predictor of change in knee flexion at mid-stance.

## Results

A total of 48 participants were initially recruited as previously described ^4^ and of those, 27 participants had a minimum of two years (mean 3.29±0.49 years) between their first and last assessment and were included in the current analysis. As per the inclusion/exclusion criteria, none of the participants had orthopaedic surgery within one year of initial analysis. Six participants had previous single-event multi-level surgery (defined as having at least two orthopaedic surgeries at a single surgical event ^22^) at a mean age of 10.03±2.60 years and a mean of 3.97±2.64 years prior to initial assessment. Thirteen participants progressed to surgical intervention without having the minimum of two years between first and last analysis while the remaining eight participants were lost to follow up or did not complete the required assessments.

**Figure 1.**
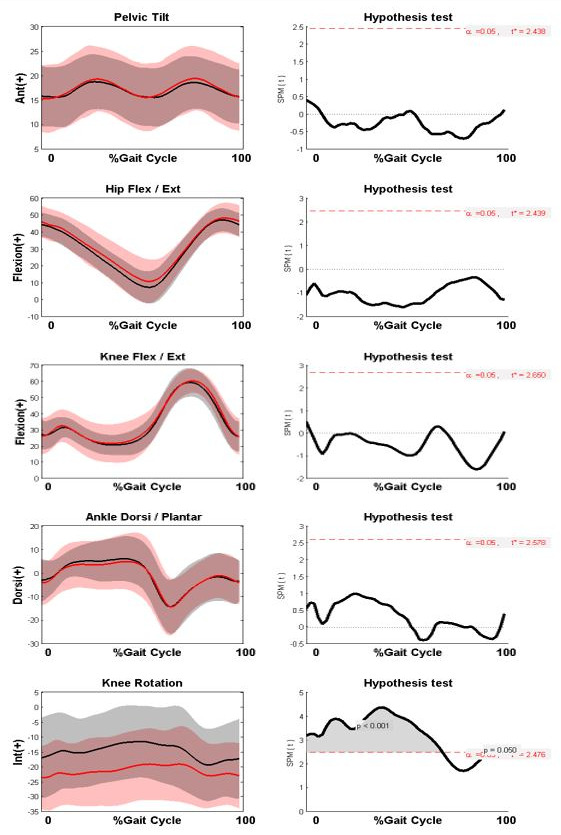
Comparison of gait kinematics at baseline and last assessment (n = 27) and subsequent SPM tests for the kinematic curves. Kinematic graphs show Baseline assessment (Black), Last assessment (Red). Shaded grey areas on SPM curves to the right highlight the period of the gait cycle where the kinematic curves differed significantly.

The participant characteristics for the 27 participants are summarised in Table 1 below. There was no significant change in mean knee flexion at mid-stance in the 3.29 years between baseline and final assessment (23.46°±5.04° and 23.71°±13.01° respectively; p=0.91) and the changes in relevant kinematic graphs between the first and last analysis are shown in Figure 1.

**Table 1.** Characteristics of the included participants (n=27).

Variable	
Age at first assessment (years)	10.43 (3.82)
Age at last assessment (years)	13.72 (3.97)
Time between analyses (years)	3.29 (0.49)
GMFCS levels I, II, III (n (%))	I-12 (44%), II-13 (48%), III-2 (7%)
Sex (M:F) (n (%))	M-16 (59%): F-11 (41%)

Values presented as mean (standard deviation). GMFCS-Gross Motor Function Classification System

There was no significant change in any sagittal plane kinematics, including knee flexion, between the first and last assessment. However, the transverse plane graphs highlight a significant increase in external knee rotation during stance phase (p<0.001). There were no significant changes in any other kinematic graphs and full kinematic graphs are shown in the Extended Data (Figure S.1).

Table 2 summarises the changes in relevant clinical examination variables. Despite no significant increase in crouch gait, hamstring tightness increased significantly (from 60.00° to 70.48°, p<0.01).

**Table 2.** Changes in clinical examination variables in the included participants (n=27).

Variable (units)	First assessment	Last assessment	p-value
Gastrocnemius Tightness (°)	97.00 (6.34)	94.19 (7.35)	0.06
Soleus Tightness (°)	100.78 (6.88)	97.04 (8.57)	0.02 *
Hamstring Tightness (°)	60.00 (10.00) ^	70.48 (5.47)	<0.01 ^Φ^ *
Femoral Torsion (°)	25.37 (7.76)	23.54 (8.81)	0.20
Tibial Torsion (°)	14.48 (5.06)	15.74 (5.19)	0.31
Hip Flexion Contracture ≥5°(n (%))	1 (4%)	4 (15%)	0.16 ^#^
Knee Flexion Contracture ≥5°	1 (4%)	3 (11%)	0.30 ^#^

Values presented as mean (standard deviation) unless ^median (interquartile range). Group comparisons are paired t-tests unless ^Φ^Wilcoxon Signed-Ranks test, ^#^Pearson’s chi-squared test. *significant differences are p<0.05

While both the mean value of knee flexion at mid-stance and SPM kinematic curves highlights that there was no overall change in knee flexion between initial and final assessments, the relatively large standard deviation associated with knee flexion at mid-stance at final assessment (23.71°±13.01°) highlights that some participants increased in crouch during the study window. Therefore, relevant participant, clinical and kinematic variables are further summarised in Table 3 for those who demonstrated increased, decreased and unchanged knee flexion at mid-stance over time. A threshold of 5° was chosen to represent a true change in the value of knee flexion at mid-stance ^23^.

**Table 3.** Summary of relevant initial and final participant, clinical examination and kinematic variables for groups demonstrating Increased (>5°), Decreased (>5°) and Unchanged (≤ 5°) crouch between initial and final assessment.

Variable (units)	Increased crouch (>50); n=7	Unchanged crouch ((≤ 50); n=13	Decreased crouch (>50); n=7
Change in knee flexion at mid-stance	10.00 (9.50) ^	-3.50 (7.00) ^	-10.00 (7.00) ^
GMFCS Levels I, I, III (n (%))	I-3(43%)	I-7(54%)	I-1(14%)
	II-3(43%)	II-6(46%)	II-5(71%)
	III-1(14%)	III-0(0%)	III-1(14%)
Sex (M: F) (n (%))	M-6(86%): F-1(14%)	M-6(46%): F-7(54%)	M-3(43%): F-4(57%)
Age at first assessment (years)	11.31 (4.01)	10.82 (3.92)	8.55 (3.61)
Time between analyses (years)	3.24 (0.58)	3.38 (0.44)	3.18 (0.51)
Initial knee flexion at mid-stance (°)	24.29(4.15)	21.00 (8.00)^	21.00 (5.00)^
Initial Hamstring Tightness (°)	63.57 (5.56)	57.53 (8.10)	54.71 (15.18)
Change in Hamstring Tightness (°)	13.00 (6.55)	14.57 (5.74)	12.43 (13.83)
Initial Tibial Torsion (°)	13.86 (6.49)	14.57 (5.74)	12.00 (2.00)^
Change in Tibial Torsion (°)	11.50 (16.54)	7.67 (7.83)	5.86 (13.21)

Values presented as mean (standard deviation) unless ^median (interquartile range). GMFCS- Gross Motor Function Classification System

Table 3 confirms that the largest group of participants (48%, n=13) did not show a significant change in knee flexion at mid-stance while smaller, equal groups (26%, n=7) demonstrated increase and decrease in crouch over time. While group sizes are small, this data might suggest that those who increased in crouch were older at initial assessment, had tighter hamstrings at initial assessment and had a larger increase in Tibial Torsion compared to the groups who either demonstrated a decrease in crouch gait or did not change. However, the association between change in crouch gait and these variables is summarised in Table 4. Regression analysis using relevant clinical examination variables as predictors of change in knee flexion at mid-stance found no significant associations.

**Table 4.** Summary of linear regression equations using clinical examination variables as predictors of change in knee flexion at mid-stance.

Predictor Variable (units)	Regression co-efficient	Confidence Interval	r ^2^	p-value
Age at first assessment (years)	0.28	-1.04, 1.59	0.01	0.67
Initial knee flexion at mid-stance (°)	0.08	-1.11, 1.26	<0.01	0.86
Initial Hamstring Tightness (°)	0.45	-0.02, 0.91	0.14	0.06
Initial Tibial Torsion (°)	0.15	-0.84, 1.15	<0.01	0.75

## Discussion

The aim of this study was to prospectively examine the progression of CP crouch gait along with the contemporaneous changes in clinical examination measures of muscle tightness and bony torsions and to examine any association between the change in crouch gait and these variables. Despite no overall change in the mean value of crouch gait in the 3.29 years between initial and follow-up analyses, there was a significant increase in mean clinical examination measure of hamstring tightness and knee eternal rotation during gait suggesting that neither of these variables contribute to crouch progression. Further regression analysis confirmed this and found that clinical examination variables were not associated with change in crouch gait. This prospectively confirms that tightening of the hamstrings does not contribute to crouch progression, is part of the natural history of children/adolescents with CP and occurs over time.

SPM analysis found no change in the majority of gait kinematic graphs, including knee flexion/extension, over two analyses 3.29 years apart. This was apart from an increase in knee rotation. Increasing external knee rotation during gait is often assumed to be part of the natural history of gait in CP and while a recent retrospective review of hamstring surgery in CP ^24^ reported similar in a small non-surgical control group (n=15), this is the first study to prospectively document this. Known issues in reliability of transverse plane kinematic data must be considered when interpreting these results. However, our laboratory has demonstrated good levels of reliability of knee transverse plane kinematics (~2.8°) ^25^ and we are confident in reporting an increase over time.

External rotation of the tibia is thought to be associated with LAD which in turn contributes to crouch. Despite the change in dynamic knee rotation evident on the kinematic graphs, the present results did not demonstrate a similar increase in mean clinical measure of tibial torsion over the same time period. Longer term prospective study might ascertain if the clinical measure of tibial torsion also increases in time and as above, a more detailed foot model might establish the potential role of foot posture contributing to the reported increased dynamic knee rotation.

Perhaps as significantly, our results highlight that reporting mean data only does not capture the significant variability in CP gait data and can hide potentially important findings. There was no change in either the mean value of knee flexion at mid-stance or the overall mean knee flexion/extension kinematic curves but there was significant variability within the overall group. While the majority of the 27 participants demonstrated unchanged knee flexion at mid-stance (n=13; 48%), equal proportions demonstrated both significant increase (n=7; 26%) and decrease (n=7; 26%) in this kinematic variable. This highlights the need to examine, and report, data in detail in studies on gait in CP to reflect the heterogeneity of this cohort and suggests that statistical analysis based on mean changes only may not sufficiently capture change. Table 3 provides a further summary of these groups. The mean values suggest that those who demonstrated an increase in crouch gait had slightly older age and tighter hamstrings at baseline compared to those who demonstrated unchanged or decreased crouch though each group demonstrated a similar increase in tightness in the hamstrings over time regardless of the changes in knee flexion at mid-stance. However, it must be highlighted that the numbers in these groups are small (n=7, 7 and 13) and more robust regression analysis on the whole study cohort found no association between progression in knee crouch and hamstring tightness or age.

It is notable that the mean value of knee flexion at mid-stance was in excess of 20° at baseline. This value has been suggested as a risk factor for accelerated progression of crouch and as a potential indicator for surgery ^3^. Based on these prospective findings, a value of 20° knee flexion at mid-stance cannot necessarily be taken as an indicator for likely progression of crouch gait and the variability of crouch progression seen in our data confirms this is a more nuanced issue.

There are some limitations and suggestions for further study that should be considered when interpreting the results of these results. This was a prospective assessment of a cohort who were already in crouch at baseline. Therefore, we cannot comment of how crouch gait initially developed in this population and likewise do not know if clinical hamstring tightness and knee external rotation during gait increase over time in a non-crouch population. The study participants were primarily GMFCS levels I and II and only two participants were GMFCS level III. This means the results of this study can only be generalised to the more functional CP population (GMFCS I and II). The time period between the first and last assessments (mean 3.3 years) is still relatively short though as our results suggest, participants with ambulant, bilateral CP will likely undergo single event multi-level surgery or other surgical intervention during the course of a prospective study making long term prospective follow up of natural progression difficult. Our results show that while the mean value of crouch did not change, there was variability within the group which may be lost when examining group means only. As is the case in many studies on CP gait, non-surgical interventions (botulinum toxin, orthoses, therapy interventions) was not considered here and it may be that these interventions play a role in the variability of gait progression and should be carefully considered in future studies. Likewise, the potential role of foot posture in the progression of crouch gait ^26^ was not fully examined in the current study and our data collection protocol did not include a multi-segment foot model.

## Conclusions

Our results further highlight the need for larger, prospective studies of gait development in CP. We would suggest that a future prospective study beginning at the onset of gait with routinely scheduled follow-up assessments not dependent on clinical referral would be extremely useful and worthwhile. A study beginning at a young baseline age would allow the development of many pathological gait patterns and associated clinical examination variables to be examined while regularly scheduled research assessments would remove any bias in terms of clinical gait analysis potentially being primarily in response to a deterioration or clinical need.

## Data Availability

Zenodo. A prospective assessment of gait kinematics and related clinical examination measures in cerebral palsy crouch gait. O'Sullivan, Rory. (2022). DOI: https://doi.org/10.5281/zenodo.7357268 This project contains the following underlying data: Data Share file. (Full relevant Underlying Data) Supplementary Figures and Table Data are available under the terms of the **Creative Commons Zero "No rights reserved" data waiver** (CC0 1.0 Public domain dedication).
