# Supplementary material for: A prospective assessment of gait kinematics and related clinical examination measures in cerebral palsy crouch gait
Source: HRB Open Res. 2023 Aug 7;5:81. Originally published 2022 Dec 6. [Version 2] doi: 10.12688/hrbopenres.13647.2 (PMC10435924; doi:10.12688/hrbopenres.13647.2)
Supplement: Peer review history

# Peer review: Reviewer response for version 2

**DOI:** 10.21956/hrbopenres.15082.r35673

**Published:** 2023-08-17

**Authors:** Hans Kainz

**Affiliations:** 1 University of Vienna, Vienna, Austria

The authors addressed all my concerns.

 I am happy with this version of the manuscript.

 The paper will be of great interest for the clinical community.

Is the work clearly and accurately presented and does it cite the current literature?

Yes

If applicable, is the statistical analysis and its interpretation appropriate?

Yes

Are all the source data underlying the results available to ensure full reproducibility?

Partly

Is the study design appropriate and is the work technically sound?

Yes

Are the conclusions drawn adequately supported by the results?

Yes

Are sufficient details of methods and analysis provided to allow replication by others?

Partly

Reviewer Expertise:

gait analysis; cerebral palsy; musculoskeletal modelling

I confirm that I have read this submission and believe that I have an appropriate level of expertise to confirm that it is of an acceptable scientific standard.

# Peer review: Reviewer response for version 1

**DOI:** 10.21956/hrbopenres.14923.r34696

**Published:** 2023-07-07

**Authors:** Andrew J. Ries

**Affiliations:** 1 Gillette Children's Specialty Healthcare, Saint Paul, Minnesota, USA

Overall study design prospectively investigates progression (or non-progression) of crouch over time in a group of individuals diagnosed with CP.

 General Comments

 It is a bit unclear to me what the actual history is of the study group. Details critical to understanding the implications of this work should be included in this manuscript and not referenced to previous work. Missing information on recruitment pool, pre-study interventions, interventions performed during the study period (surgical and non-surgical), etc.

 The biggest methodological issue I have is with using a single trial as ‘representative’ for each visit. We know individuals diagnosed with CP often have significant stride-to-stride variability during gait. A more robust analysis would utilize all trials collected during a single session in an effort to actually quantify gait variability and more appropriately determine the likelihood of real changes in crouch on an individual basis. Personally, I don’t have a lot of confidence as to the veracity of the kinematic comparison and individual analysis of changes in crouch presented in this manuscript given this ‘single trial for analysis’ shortcoming.

 Introduction

 The main point in your introduction is that sampling bias likely led to misinterpretation as to the rate of crouch gait progression in the CP population. Please describe, in this manuscript, how the prospective sample was recruited (i.e. were these individuals drawn from a population that was already referred to the gait lab). If they were drawn from a population already referred to the gait lab, how is this prospective sample any different from those evaluated via  – as per your introduction – ‘inferior’ retrospective study designs (i.e. “ *referral for clinical gait analysis is often as a result of a deterioration in function, pain or to aid with planning of required intervention*”). If these individuals were NOT recruited from those already referred to the gait lab, can you be certain that collection/capture of gait analysis data did not alter future treatment decisions regarding crouch gait (i.e. treatment plan prior to gait lab might have changed to include different interventions based on the additional gait lab data collected as part of this study, which otherwise would not have been performed)? Even if these individuals were recruited from individuals within the hospital system, doesn’t that also suffer from the same pitfalls as you previously described for retrospective analyses (i.e. patients are often at the hospital for as a “ *result of a deterioration in function, pain or to aid with planning of required intervention*”). Finally, if the ‘prospective’ part of the study was simply to follow-up with all individuals who were initially referred to the gait lab, the applicability of these results becomes much narrower (e.g. change/no change in crouch gait applies only to those individuals undergoing standard clinical practice following referral to the gait lab, and don’t necessarily reflect any other CP population).

 Methods

 You only utilize a single ‘representative’ trial despite collecting four for each individual? Why not utilize all 4 trials to give a more robust assessment of variability at each timepoint? A rhetorical question would be: If you are only going to use one trial, why do you even bother collecting four? The significant methodological issue when using a single trial is that you don’t have the ability to quantify any inter-session variability. It’s especially problematic in a population that commonly has large stride-to-stride variability. As such, how is it possible to draw robust conclusions about improvement/worsening of crouch on an individual basis (like you go on to do) when only utilizing a single trial per visit?

 Results

 This study is framed as a ‘natural progression of crouch’, but it is not clear that this is the case. Please include all details of the study group relevant to this study rather than referring back to your previous publication. How were these individuals recruited (gait lab referral, hospital population, general population), what general procedures were performed prior to study participation, what procedures were performed during the study period, how many of these individuals have AFOs, etc.

 Related to the previous comment, in is unclear (and somewhat confusing) to me based on what is written in the first paragraph of the results section which participants were included and what types of intervention were performed at what point in time before and during the experimental time period. For example, regarding the statement “ *Thirteen participants progressed to surgical intervention without having the minimum of two years between first and last analysis while the remaining eight participants were lost to follow up or did not complete the required assessments.*”. Are these 13 included in the 27 or are they part of the 48 that weren’t included in this study? It’s also important to know what procedures (in bulk) the 27 individuals had during the course of the study.

 Were changes in knee rotation towards or away from typically developing norms? Regardless of direction, do you think changes in transverse plane rotations (both kinematic changes and linear regression modeling) provide strong enough evidence to question the belief that lever arm dysfunction has a significant effect on crouch gait?

 You mention large variability in knee flexion suggests differing group responses (worsened, same, improved). How can you be certain that this variability is true and not a consequence of only using one trial per visit (“ *one representative walking trial was chosen for further analysis*”). In other words, these results could simply be caused by trial-to-trial gait variability (e.g. picked a single trial that happened to be the high end relative to the others for visit 1 and a trial that happened to be on the low relative to the others for visit 2, while overall mean values of all trials for both visits are the same).

 Discussion

 Related to previous comment, the “ *natural history*” statement in “ *This prospectively confirms that tightening of the hamstrings does not contribute to crouch progression, is part of the natural history of children/adolescents with CP and occurs over time.*” implies that there were no treatments provided to any of these individuals during the study period. A purist might even say that any treatment provided at any time to these individuals (even prior to recruitment to this study) should prohibit the use of ‘natural history’ since any intervention *might* have the potential to alter body development. Please explicitly list out what general interventions this sample population underwent prior to and during the study period. This would help readers better understand the context in which to evaluate these results and conclusions.

 You mention 20 deg is a commonly utilized ‘tipping point’ for worsening of crouch, and you concluded from the results of this study that it doesn’t necessarily mean that this is the case. I understand that you only studied individuals with significant crouch, but I wonder if future studies wouldn’t benefit from looking at crouch in CP more broadly, especially considering the high rate of crouch gait in CP, in general. Instead of 20 deg of crouch being a tipping point where PE and kinematic changes start to occur, maybe physiological changes begin to occur much earlier, say from 0-10 deg of crouch, and then don’t really change after that. My point is, why not focus on the bigger picture, what causes crouch or what changes occur during early onset of crouch rather than hyper-focusing on what causes bad crouch to get worse.

 You state that “our results highlight that reporting mean data only does not capture the significant variability in CP gait data and can hide potentially important findings.” But isn’t this also true for single trial data (i.e. ‘Reporting single trial data does not capture the significant variability in CP gait data and can hide potentially important findings.’)? I don’t argue with the potential that sub-groups can exist that have different responses than the overall group, but it seems naïve to pretend that comparing single gait trials at two time points gives a robust picture of gait kinematics, especially considering the large stride-to-stride variability often observed in individuals diagnosed with CP.

Is the work clearly and accurately presented and does it cite the current literature?

Yes

If applicable, is the statistical analysis and its interpretation appropriate?

Partly

Are all the source data underlying the results available to ensure full reproducibility?

Yes

Is the study design appropriate and is the work technically sound?

Partly

Are the conclusions drawn adequately supported by the results?

Yes

Are sufficient details of methods and analysis provided to allow replication by others?

Partly

Reviewer Expertise:

gait analysis, cp, musculoskeletal modeling, gait energetics

I confirm that I have read this submission and believe that I have an appropriate level of expertise to confirm that it is of an acceptable scientific standard, however I have significant reservations, as outlined above.

# Peer review: Reviewer response for version 1

**DOI:** 10.21956/hrbopenres.14923.r34703

**Published:** 2023-07-07

**Authors:** Hans Kainz

**Affiliations:** 1 University of Vienna, Vienna, Austria

This is a well written paper with some interesting findings. Please find below my detailed feedback.

 Introduction is well written and provides a good summary of previous research related to the topic of this paper.

 Last sentence in the introduction: Please change the wording from 'measures of bony torsions' to 'estimates of bony torsions'. Otherwise the reader expects that you used medical images to quantify bony torsions.

 Methods:

 First paragraph in the method section: Please clearly state that data of the same participants has been published in your previous paper. 

 How did you choose the representative walking trial for your analysis? Was it based on a qualitative evaluation of the kinematic waveforms or did you use a quantitative measure?

 Please specify which SPM tests you used for your analyses.

 Results:

 Figure 1: I know the additional figures are in the supplementaty material but I believe it would be better to report pelvic, hip and knee angles in all three planes in the main manuscript.

 Figure 1: Hypothesis test waveforms take a lot of space and could be included in the kinematic waveform subplot, e.g. horizontal bars indicating significant differences below the kinematic waveforms. From my point of view it would make the figure nicer. However, this is my own preference and I leave it up to the authors to decide how they will present the SPM results in this figure.

 Table 2: Why is hamstring tightness from the first assessment reported as median (interquartile range) while the last assessment is presented as mean (standard deviation)? Please use the same method to make it easier for the reader to compare both assessments.

 Table 3: Label at the top is wrong. '>50' should be '>5°', etc. Furthermore, having the same numbers in brackets for increased and decreased crouch was a bit confusing for me. Might be better to report the numbers in brackets as >5°, +/-5° and <5°.

 Furthermore, the authors could consider to perform statistical test between these 3 groups.

 In general, I believe the additional analyses are interesting. I, however, suggest to mention the additional analyses in the method section.

 Discussion:

 Third paragraph: Further thought from my side which might be worthwhile mentioning: Clinical measures of bony torsion are not very reliable. I would recommend long term prospective studies based on medical images, which enable a more reliable and accurate estimation of bony torsion.

 Fourth paragraph: Very interesting findings. Change in tibia torsion was larger in the increased crouch gait group which might be worthwhile to mention/discuss in this paragraph.

 The lack of medical images to quantify bony torsion is another limitation of this study.

 Conclusion:

 I agree with the content of this paragraph, but I would have expected that the conclusion paragraph is more related to the findings/data from this study.

 Are all the source data underlying the results available to ensure full reproducibility?

 I answered 'partly' to this question because the raw data, i.e. c3d files from the motion capture data are not provided. However, this is common within the communuity and I do not have a problem with it.

Is the work clearly and accurately presented and does it cite the current literature?

Yes

If applicable, is the statistical analysis and its interpretation appropriate?

Yes

Are all the source data underlying the results available to ensure full reproducibility?

Partly

Is the study design appropriate and is the work technically sound?

Yes

Are the conclusions drawn adequately supported by the results?

Yes

Are sufficient details of methods and analysis provided to allow replication by others?

Partly

Reviewer Expertise:

gait analysis; cerebral palsy; musculoskeletal modelling

I confirm that I have read this submission and believe that I have an appropriate level of expertise to confirm that it is of an acceptable scientific standard, however I have significant reservations, as outlined above.

# Peer review: Reviewer response for version 1

**DOI:** 10.21956/hrbopenres.14923.r33258

**Published:** 2023-01-03

**Authors:** Erika Cloodt

**Affiliations:** 1 Department of Clinical Sciences Lund, Orthopaedics, Lund University, Lund, Sweden

This study aims to prospectively analyse the association between clinical variables and change in crouch gait. 

 I have some comments and questions to the authors: 

 Abstract:

- Conclusion: Does not answer to your aim to examine the association between crouch and clinical variables. And are the problems only with the heterogeneous population? Or your small study population of 27 individuals?

Introduction:

- Please clarify why it's important to examine associated changes in clinical examination and crouch? Add references to how those variables are associated with crouch. 

Methods:

- You refer to your previous study for description of data collection. In that study- the participants had an examination every 6 months: was that the case also for this study? Please clarify this. 
- You used two SD or more as a definition of crouch. In your previous study - you did not consider ankle kinematics and therefore referred to "flexed knee gait" instead of crouch. As far as I can tell, you used the same participants in this study - So how do you explain the different use of "crouch" definition?

Results: 

- I miss a summary of level of crouch at first assessment.
- Table 3: Parentheses in table heading should be (<5 >5 etc and not <50)

Discussion

- Needs to be improved by comparing your results with other relevant articles and the clinical relevance of it. See Kanashivili and Church for example. 
- You used 2 SD as a definition of crouch. Kanashvili et al. 2021 and Church et al. 2018 both defined 1-3 SD as mild crouch, 3-5 as moderate and >5 as severe. Could this have affected your results? What was the reason for using 2 SD based on previous studies? Please address. 
- Is there any difference in level of decrease or increase of crouch based on the level at first assessment? Potentially - if you already walk with severe crouch - is it less likely to increase further and still be able to walk?
- You examines all individuals barefoot. Many individuals walking in crouch are using orthoses to gain a better walking performance - why examine them barefoot if this is not their normal way of walking? Please discuss.  
- Did the participants receive any specific interventions to improve gait between assessments? If they were referred to a gait lab, is it likely that their coming interventions were based on the results of the analysis? Please discuss this and how it might affect the results. 

Conclusion:

- Does not answer to your aim to analyse the association between clinical variables and crouch. You found no association?

References:

 Kanashvili B, Miller F, Church C, Lennon N, Howard JJ, Henley JD, Niiler T, Sees JP, Rogers KJ, Shrader MW. The change in sagittal plane gait patterns from childhood to maturity in bilateral cerebral palsy. Gait Posture. 2021 Oct;90:154-160. doi:10.1016/j.gaitpost.2021.08.022. ^1^ 

 C. Church, J. Ge, S. Hager, T. Haumont, N. Lennon, T. Niiler, et al., Flexed-knee gait in children with cerebral palsy: a long-term follow-up study, Bone Joint J. 100- B (2018) 549–556, https://doi.org/10.1302/0301-620X.100B4.BJJ-2017-0732.R1. ^2^

 J.M. Rodda, H.K. Graham, L. Carson, M.P. Galea, R. Wolfe, Sagittal gait patterns in spastic diplegia, J. Bone Joint Surg. Br. 86 (2004) 251–258, https://doi.org/ 10.1302/0301-620x.86b2.13878. ^3^

Is the work clearly and accurately presented and does it cite the current literature?

Partly

If applicable, is the statistical analysis and its interpretation appropriate?

Partly

Are all the source data underlying the results available to ensure full reproducibility?

Yes

Is the study design appropriate and is the work technically sound?

Yes

Are the conclusions drawn adequately supported by the results?

Partly

Are sufficient details of methods and analysis provided to allow replication by others?

Partly

Reviewer Expertise:

Cerebral palsy, contractures, othopedics.

I confirm that I have read this submission and believe that I have an appropriate level of expertise to confirm that it is of an acceptable scientific standard, however I have significant reservations, as outlined above.

## References

[1] : The change in sagittal plane gait patterns from childhood to maturity in bilateral cerebral palsy. *Gait Posture* .2021;90: 10.1016/j.gaitpost.2021.08.022 154-160 10.1016/j.gaitpost.2021.08.022 34481266

[2] : Flexed-knee gait in children with cerebral palsy: a long-term follow-up study. *Bone Joint J* .2018;100-B(4) : 10.1302/0301-620X.100B4.BJJ-2017-0732.R1 549-556 10.1302/0301-620X.100B4.BJJ-2017-0732.R1 29629589

[3] : Sagittal gait patterns in spastic diplegia. *J Bone Joint Surg Br* .2004;86(2) : 10.1302/0301-620x.86b2.13878 251-8 10.1302/0301-620x.86b2.13878 15046442
